# Template-free fabrication of silicon micropillar/nanowire composite structure by one-step etching

**DOI:** 10.1186/1556-276X-7-557

**Published:** 2012-10-08

**Authors:** Fan Bai, Meicheng Li, Rui Huang, Dandan Song, Bing Jiang, Yingfeng Li

**Affiliations:** 1 State Key Laboratory of Alternate Electrical Power System with Renewable Energy Sources, North China Electric Power University, Beijing, 102206, China; 2 School of Materials Science and Engineering, Harbin Institute of Technology, Harbin, 150001, China; 3 Suzhou Institute, North China Electric Power University, Suzhou, 215123, China

**Keywords:** Micropillars, Nanowires, Metal-assisted electroless etching, K_2_SiF_6_

## Abstract

A template-free fabrication method for silicon nanostructures, such as silicon micropillar (MP)/nanowire (NW) composite structure is presented. Utilizing an improved metal-assisted electroless etching (MAEE) of silicon in KMnO_4_/AgNO_3_/HF solution and silicon composite nanostructure of the long MPs erected in the short NWs arrays were generated on the silicon substrate. The morphology evolution of the MP/NW composite nanostructure and the role of self-growing K_2_SiF_6_ particles as the templates during the MAEE process were investigated in detail. Meanwhile, a fabrication mechanism based on the etching of silver nanoparticles (catalyzed) and the masking of K_2_SiF_6_ particles is proposed, which gives guidance for fabricating different silicon nanostructures, such as NW and MP arrays. This one-step method provides a simple and cost-effective way to fabricate silicon nanostructures.

## Background

Silicon nanostructures, including silicon nanohole, silicon nanowire, and silicon nanopillar, have attracted wide attention due to their potential in various fields of application, such as solar cells [1-3], lithium batteries [4], insulator transistors [5], and gas and chemical sensors [6,7]. In particular, silicon micropillar (MP)/nanowire (NW) composite structure becomes more interesting recently due to its excellent light trapping and efficient carrier collection, which is applied to design and construct high performance radial p-n junction solar cells [8,9]. At present, the effective fabrication method for silicon MP/NW structure is the wet or dry etching of silicon substrate combined with various templates, such as silica dot array [8] and circular-shaped photoresist dots [9,10]. However, the use of these templates increases the fabrication cost and also makes the manufacturing process complicated. Therefore, searching a simple and cost-effective fabrication approach for silicon MP/NW structure is necessary.

Recently, K_2_SiF_6_ crystallites have been observed during the different wetting etchings of silicon substrate in the presence of HF and K^+^ ions, such as chemomechanical polishing [11], stain etching [12], laser-assisted etching [13], and metal-assisted electroless etching (MAEE) [14,15]. These K_2_SiF_6_ crystallites are insoluble in dilute HF solution and to some extent can prevent the etchant solution from the contact with silicon surface. Therefore, K_2_SiF_6_ crystallites offer a possible mask approach to selectively remove silicon materials. Previous studies focus on the effect of K_2_SiF_6_ crystallites on the formation of porous silicon and its photoluminescence property, but utilizing K_2_SiF_6_ as a template to fabricate silicon nanostructures has not been studied.

In this work, a template-free fabrication method for silicon MP/NW structure is presented. By utilizing an improved MAEE of silicon in KMnO_4_/AgNO_3_/HF solution, silicon MP/NW structure was achieved under the mask of self-growing K_2_SiF_6_ particles. This simple one-step fabrication method integrates the masking process and the etching process, avoiding conventional masking procedures, which provide a simple and cost-effective route to fabricate silicon nanostructures.

## Methods

In these experiments, p-type Si(100) wafers with a resistivity around 7 to 13 Ω·cm were used. The wafer was cut into 1.5 × 1.5 cm^2^ pieces and used as test samples. Silicon samples were ultrasonically cleaned in acetone, absolute alcohol, and deionized water successively. Then, the cleaned Si samples were dipped into dilute HF solution to remove native oxide. Following the cleaning step, the etching process was performed through immersing the silicon samples into the etchant solution, which contains 5 M HF, 0.02 M AgNO_3_, and KMnO_4_ with different concentrations. The reaction time varied from 15 to 90 min. After etching process, Si samples were rinsed with deionized water and then immersed into the concentrated HNO_3_ to remove the retaining silver and other residue. All treatments were performed at room temperature.

The morphologies of the silicon nanostructures were observed by the scanning electron microscope (SEM) with FEI Quanta 200 F (FEI Company, OR, USA). Crystals covered on the Si samples were analyzed using the energy dispersive X-ray (EDX), and X-ray diffraction (XRD) by Bruker D8 Focus X-ray powder diffractometer (Bruker Corporation, MA, USA) with Cu Kα radiation (*λ* = 1.5406 Å).

## Results and discussion

K_2_SiF_6_ crystallite, spontaneously generating when the solubility product of K_2_SiF_6_ exceeded to 6.3 × 10^−7^ mol^3^dm^−9^, is a byproduct during the forming process of porous silicon [11]. Hadjersi et al. reported that an insoluble solid-phase film (K_2_SiF_6_) covered the top of porous silicon layer by the etching of silicon-coated silver film in HF-oxidizing solution [14]. Also, the existence of K_2_SiF_6_ layer causes the decrease of the etching rate of silicon [16]. These results demonstrated that K_2_SiF_6_ has an ability to form a masking layer during MACE process. Based on these, an improved MACE approach, integrating three processes (including the deposition of catalyzed silver nanoparticles (NPs), the formation of K_2_SiF_6_ mask, and the electroless etching of silicon) was utilized to realize one-step fabrication of silicon nanostructures.

**Figure 1:**
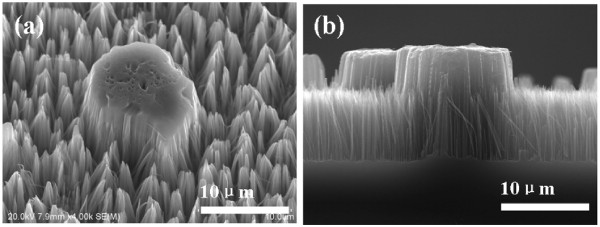
**SEM images of silicon MP**/**NW structure.** (**a**) Plane-view and (**b**) cross-sectional SEM images of silicon MP/NW structure obtained from the etching of silicon in the solution containing 4.6 M HF, 0.02 M AgNO_3_, and 0.05 M KMnO_4_ for 45 min.

Using this thinking, through a systematic process optimization, the silicon MP/NW composite structure was achieved by the simple template-free method. Figure 1 shows the silicon nanostructure obtained with the etching solution containing 4.6 M HF, 0.02 M AgNO_3_, and 0.05 M KMnO_4_. Both micropillars and nanowires can be observed on the whole silicon substrate, and every pillar is surrounded by the silicon NWs array closely, implying that the silicon MP/NW composite nanostructure can be realized by the one-step etching process. Meanwhile, we found that, in the silicon MP/NW composite structure, there are a lot of obvious differences between MPs and NWs. Firstly, the distribution of the silicon MPs is discrete and heterogeneous, whereas that of NWs is dense and homogeneous (as shown in Figure 1a). Secondly, the length of MPs and NWs is also quite different, that is, 13.5 μm for MPs and 8.4 μm for NWs (as shown in Figure 1b). Furthermore, the shape of these silicon MPs is approximately circular, and the diameter is in a range from several micrometers to dozens of micrometers, which is much larger than the diameter of 30 to 300 nm for the silicon NWs. Compared to the single type of silicon nanostructures (i.e., the thick cylindrical nanopillars and the order nanowires array) prepared by conventional etching process [17-19], the differences of the characteristics suggest that the formation process of the silicon MP/NW composite structure is complicated significantly.

**Figure 2:**
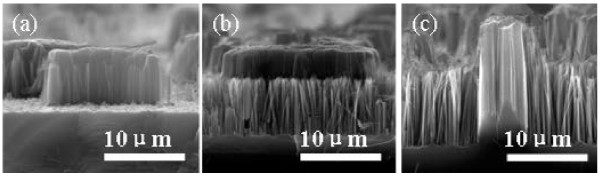
**Cross**-**sectional SEM images.** Silicon samples are etched in the solution containing 4.6 M HF, 0.02 M AgNO_3_, and 0.05 M KMnO_4_ for different reaction times. (**a**) 15 min; (**b**) 45 min; (**c**) 90 min.

To disclose the formation process of silicon MP/NW composite structure in the HF/AgNO_3_/KMnO_4_ solution clearly, the morphology evolution on the surface of etched silicon with reaction time was investigated. At initial stage of the chemical etching reaction (less than 15 min), the silicon MPs and few silicon NWs were observed on silicon substrate, as shown in Figure 2a. Then, as reaction time increase, the length of MPs gradually increases and NWs with a short length closely generate around these MPs, as shown in Figure 2b. Meanwhile, MPs are lightly etched at their top surface and sidewalls. With the further increasing of reaction time, the lengths of the silicon MPs and the silicon NWs continually increase, whereas the silicon MPs were etched significantly on their sidewalls, resulting in the damage of the silicon MPs (as shown in Figure 2c). These results indicate that the formation of the silicon MPs is prior to the formation of the silicon NWs. Therefore, to get the silicon MP/NW composite structure, there should be a suitable range of the reaction time.

**Figure 3:**
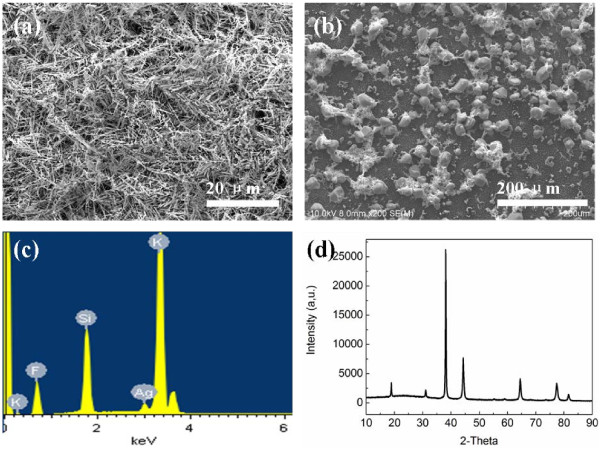
**Surface morphology.** The silicon sample etched in the solution containing 4.6 M HF, 0.02 M AgNO_3_, and 0.05 M KMnO_4_ for 45 min (**a**) before cleaning by deionized water and (**b**) after cleaning by deionized water. (**c**) EDX spectrum and (**d**) XRD spectra corresponding to (b), respectively.

The simultaneous emergence of the silicon MPs and the silicon NWs on silicon substrate here must be associated with the etching in the case of using the mask. In this work, K_2_SiF_6_ crystalline formed during the etching process should be the most possible template, so its existence and role in the formation process of silicon MP/NW structure were investigated systematically. Figure 3 displays the morphology and the chemical composition of the gray layer covering on the surface of silicon samples without removing residue on the silicon surface by HNO_3_ just after the etching process. From Figure 3a, it can be seen that the top surface of the silicon substrate is covered by a layer of loose silver dendrites, which plays an important role on the formation of NW array [20]. After the removal of silver dendrites by the rinse of deionized water, a number of spherical-shaped particles were clearly observed on silicon substrate, as shown in Figure 3b. The analysis of EDX spectrum displays that the main components of these particles are potassium (K), silicon (Si), fluorine (F), and silver (Ag) (Figure 3c). Furthermore, as shown in Figure 3d, the diffraction peaks from XRD spectra are in agreement with the characteristic peaks of K_2_SiF_6_ reported by Hadjersi [15] and Loehlin et al. [21] (Figure 3d). These results confirm that these particles are composite of K_2_SiF_6_. Notably, we can see that these K_2_SiF_6_ particles are about dozens of micrometers in diameter, which is in accordance with the diameter of the silicon MPs. It implies that K_2_SiF_6_ particles spontaneously form the templates during the etching process, further leading to the formation of the silicon MPs.

**Figure 4:**
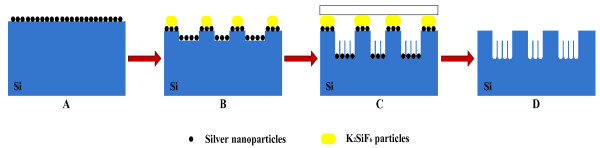
**Formation mechanism of MP and NW arrays through the one**-**step etching in HF**/**AgNO**_**3**_/**KMnO**_**4**_**solution.**

Based on the above analyses, a formation mechanism of the silicon MP/NW composite nanostructure through the one-step etching in HF/AgNO_3_/KMnO_4_ solution was proposed. First of all, when the silicon wafer was immersed into the HF/AgNO_3_/KMnO_4_ solution, many Ag NPs deposit on silicon substrate via the electroless deposition process (as described in Figure 4A), forming a number of nanoscale electrochemical cells at Ag NPs/Si areas [18]. These Ag NPs act as reaction cathodes, while the silicon areas underneath Ag NPs act as reaction anodes. At reaction cathodes, besides the reductive deposition of silver, MnO_4_^−^ reacts with H^+^ ions and generates a large number of holes, which inject into the silicon with a mediate of Ag NPs. The reaction equation is described as follows:

(1) $$Ag^{+}+e\to Ag$$

(2) $$MnO_{4^{-}}+8H^{+}\to Mn^{2+}+4H_{2}O+5{\text{h}}^{+}$$

Meanwhile, at reaction anodes, silicon areas injected by holes are oxidized and further dissolved in HF solution, causing the formation of SiF_4_, which can be easily hydrolyzed to SiF6^2−^. The reaction equation is written as follows:

(3) $$Si+4{\text{h}}^{+}+6HF\to H_{2}SiF_{6}+4H^{+}$$

At initial stage of etching, since the standard reduction potential of MnO4^−^ (1.51 eV) is larger than that of Ag (0.78 eV) [22], injected holes are provided mainly from S2. As the reactions (S2 and S3) continuously proceed, the concentration of SiF6^2−^ increases gradually. When the concentration of SiF6^2−^ is accumulated sufficiently, K_2_SiF_6_ can heterogeneously nucleate at the silicon surface and grow up to K_2_SiF_6_ particles, covering dense silver NPs (as described in Figure 4B). So, K_2_SiF_6_ particles shelter parts of Ag NPs and further prevent the etchant solution from the contact with Ag NPs. It is difficult for hole injection from Ag NPs to silicon areas covered by K_2_SiF_6_ particles. At the same time, at the areas of silicon surface without K_2_SiF_6_ particles, silicon is still subjected to the etching assisted by the catalysis of Ag NPs. Therefore, the silicon under K_2_SiF_6_ particles was retained, while the silicon not covered with K_2_SiF_6_ particles was etched away, leading to micropillar structure on the silicon substrate. As the reaction continuously performs, the concentration of MnO4^−^ in the solution is reducing; injected holes are provided mainly by S1. On the surface of silicon without K_2_SiF_6_ particles covered, nanowire array form closely around micropillars and simultaneously accompanied the deposition of silver dendrites (as described in Figure 4C), which is clearly illustrated by the formation model of SiNW array in HF/AgNO_3_ solution [2,23]. Finally, the silicon MP/NW composite structure was obtained after the cleaning by HNO_3_, as described in Figure 4D.

**Figure 5:**
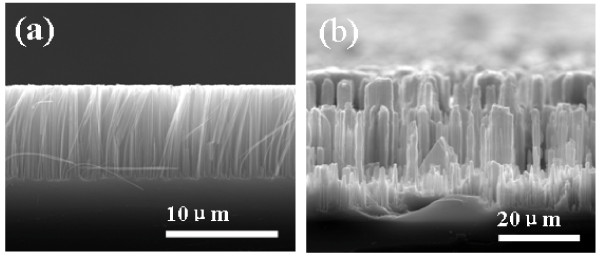
**Cross**-**sectional SEM images of silicon samples.** (**a**) Etched in the solution containing 4.6 M HF, 0.02 M AgNO_3_, and 0.005 M KMnO_4_ for 45 min. (**b**) Cross-sectional SEM images of n-Si(100) with the resistivity of 3 cmto 5 Ω·cm etched in the solution containing 4.6 M HF, 0.02 M AgNO_3_, and 0.05 M KMnO_4_ for 45 min.

According to experimental results and mechanism analysis, it can be seen that utilizing K_2_SiF_6_ particles as a template to fabricate the silicon MP/NW composite structure is a feasible method. If the size and the amount of K_2_SiF_6_ particles formed on silicon substrate could be controlled, this simple fabrication method for the silicon nanostructures will have greater practical application value. Thus, many process parameters such as KMnO_4_ concentration, the type of silicon wafer, and reaction temperature were adjusted to modulate the homogeneous distribution of K_2_SiF_6_ particles and to prepare different silicon nanostructures. For example, ordered silicon nanowire arrays were achieved at low concentration of KMnO_4_ (i.e., 0.005 M), as shown in Figure 5a. Moreover, uniform silicon pillar array was also achieved when silicon wafer with small resistivity (i.e., 3 to 5 Ω·cm) was etched in the same solution, as shown in Figure 5b. Thus, this simple fabrication process will be an effective approach to fabricate a variety of silicon nanostructures.

## Conclusion

A simple fabrication method integrating the masking process of K_2_SiF_6_ particles and the silver-assisted electroless etching process is presented to fabricate silicon nanostructures. Using this method, silicon MP/NW composite structure was successfully fabricated, and their lengths can be controlled by adjusting reaction parameters. By the observation of EDX and XRD, it is demonstrated that the electroless etching under the mask of K_2_SiF_6_ particles causes the formation of silicon MP/NW structure. Further, a formation mechanism of the silicon MP/NW composite structure in KMnO_4_/AgNO_3_/HF solution was proposed. Based on these, different silicon nanostructures such as nanowire and pillar arrays can also be achieved by adjusting the size and distribution of K_2_SiF_6_ particles.

## Competing interest

The authors declare that they have no competing interests.

## Authors' contributions

FB did the most of the experiments and drafted the manuscript. ML designed the research idea, figured out, and rewrote the paper. RH did part of the research experiments. DS participated in the design of the study. BJ and YL took part in the discussion of the research. All authors read and approved the final manuscript.

## Authors' information

FB, a Ph.D. candidate, is under the supervisory of Prof. ML. He obtained his masters degree at the Wuhan University of Technology in 2010. At present, his research interests are as follows: silicon-based solar cells, fabrication of large-areas graphene, and light trapping silicon surface structure. ML is a professor in Renewable Energy and Clean Energy. He is the director of New Energy Materials and PV Technology Center, State Key Laboratory of Alternate Electrical Power System with Renewable Energy Sources. He worked in the University of Cambridge as a research fellow from 2004 to 2006. His expertise is in the fields of design and fabrication of energy materials, functional micro-nanostructures, and energy conversion devices.
